# Community Caregivers’ Perspectives on Health IT Use for Children With Medical Complexity: Qualitative Interview Study

**DOI:** 10.2196/67289

**Published:** 2025-02-10

**Authors:** Farah Elkourdi, Onur Asan

**Affiliations:** 1 Department of Systems and Enterprises Stevens Institute of Technology Hoboken, NJ United States

**Keywords:** pediatric care, children with medical complexity, family-centered care, health information technology, health care software solutions, mobile phone, artificial intelligence

## Abstract

**Background:**

Children with medical complexity represent a unique pediatric population requiring extensive health care needs and care coordination. Children with medical complexities have multiple significant chronic health problems that affect multiple organ systems and result in functional limitations and high health care needs or use. Often, there is a need for medical technology and total care for activities of daily living, much of which is provided at home by family and caregivers. Health IT (HIT) is a broad term that includes various technologies, such as patient portals, telemedicine, and mobile health apps. These tools can improve the care of children with medical complexity by enhancing communication, information exchange, medical safety, care coordination, and shared decision-making. In this study, we identified children with medical complexity as children aged <21 years who have >3 chronic health conditions. Community caregivers contribute to the care management of children with medical complexity, serving as advocates and coordinators, primary sources of information about children’s needs, and facilitators of access to care. They are often the first point of contact for the families of children with medical complexity, particularly in vulnerable communities, including families in rural areas, low-income households, and non–English-speaking immigrant populations.

**Objective:**

This study aims to introduce the HIT needs and preferences for children with medical complexity from the perspective of community caregivers. By including their perspective on HIT development, we can better appreciate the challenges they face, the insights they offer, and the ways in which they bridge gaps in care, support, and resources.

**Methods:**

We conducted semistructured interviews (n=12) with formal community caregivers of children with medical complexity populations from a parent advocacy network on the US East Coast. Interviews were audio recorded via Zoom and then transcribed. An inductive thematic analysis was conducted to reveal HIT challenges and preferences for improving the care of children with medical complexity.

**Results:**

We categorized the interview results into themes and subthemes. There are four main themes: (1) telehealth transforming care for children with medical complexity during the COVID-19 pandemic, (2) suggested tools and technologies for care for children with medical complexity, (3) HIT feature preferences, and (4) transition to adult care. Each theme had multiple subthemes capturing all details related to design features of needed technologies.

**Conclusions:**

The study emphasizes the need to develop and enhance HIT for the care of children with medical complexity. The identified themes can serve as design guidelines for designers by establishing a foundation for user-centered HIT tools to effectively support children with medical complexity and their families. Telehealth and mobile health apps could improve care management and quality of life for children with medical complexity.

## Introduction

### Background

Children with medical complexity are a unique group within the pediatric population, characterized by diverse and significant medical needs. Though they represent <1% of children in the United States, children with medical complexities face chronic health conditions that are severe and enduring [1]. These children, all aged <21 years, often have congenital or acquired multisystem diseases or severe neurological conditions that lead to substantial functional impairments [2]. Although there is no universal definition, we used the most common criteria in our study for children with medical complexity as children aged <21 years who have >3 chronic health conditions [2].

Caring for children with medical complexity requires collaboration among stakeholders, including parents, health care providers in hospitals and clinics, school and home nurses, and community caregivers. Despite efforts, the challenges in coordinating care for these children persist, remaining largely unresolved [3]. These challenges include barriers to technology systems, inadequate access to health information, and a lack of partnership in care [4]. The burden of managing these complex medical needs falls heavily on parents, who often experience a significant caregiver burden [5].

Various technologies have been used in health care to improve the quality-of-care process. Health IT (HIT), including patient portals, telemedicine, and mobile health apps, has the potential to help with data management, sharing, and care coordination [6]. These HITs can positively impact medical outcomes, including physical, psychological, and continuity of care [7], minimize medication errors, and provide safer care [8]. For instance, telehealth emerged as a primary technology to provide safer care to children with medical complexity during the COVID-19 pandemic. Children with medical complexity is a vulnerable population that can benefit from technological interventions to improve care management, including information exchange, shared decision-making, and follow-up [9]. However, there is a need for studies to evaluate the effectiveness of HIT in caring for the children with medical complexity population and to identify the challenges and limitations associated with it. It is also critical to explore strategies for safely and effectively integrating HIT into the overall care management of children with medical complexity [10-12].

While parents are responsible for the day-to-day care of their children, community caregivers play a broader role by helping parents navigate complex health care systems. In addition, they ensure that children with medical complexities become fully participating and contributing members of their communities [13]. Community caregivers contribute to the care management of children with medical complexity, serving as advocates, coordinators, primary sources of information about children’s needs, and facilitators of access to care [13]. In addition, they support families in vulnerable communities who have a child with a medical complexity, including families in rural areas, low-income households, and non–English-speaking immigrant populations [13,14]. Their experience navigating health care systems and HIT enables them to identify gaps in care and areas for improvement. Understanding their feedback and perspective on the use of technology in care for children with medical complexity is essential.

### Objective

This study aimed to fill this gap by exploring how current tools and technologies are used and how they can be further optimized to meet the specific needs of children with medical complexity from the viewpoint of community caregivers. By gathering their insights, challenges, and recommendations, this study seeks to guide the development of technological solutions that enhance communication among stakeholders and improve the quality of life for children with medical complexity and their families. To our knowledge, this is the first study to specifically address technology suggestions from the perspective of formal community caregivers.

## Methods

### Study Design and Data Collection

We recruited formal community caregivers of children with medical complexity populations from the Parent Advocacy Network organization based in New Jersey in the United States, which was founded by the parents of children with special needs in 1987 to provide support to the families of patients with special needs [13]. The staff have supported >500 families of children with medical complexity in New Jersey for many years. We disseminated an informative recruitment email to all the staff members to participate in our study. Most of the staff are also parents of patients with special needs. We used a theoretical data saturation approach in recruitment. The saturation level was determined as the stage where adding more interviews would no longer enrich the conceptual depth of existing themes or reveal additional themes [15]. We ended up with 12 in-depth, semistructured interviews conducted from April 2023 to August 2023. Most (11/12, 92%) participants were female, and (1/12, 8%) participants were male. This study has an exploratory nature to identify various suggestions and needs in HIT design requirements for care for children with medical complexity. The exploratory nature of a qualitative approach generates very rich data and allows us to capture the caregivers’ detailed experiences in the matter.

We conducted audio interviews over Zoom (Zoom Video Communications) and recorded all the interviews to be transcribed verbatim for the analysis. The interview guide included questions on the pros and cons of technologies used in care for children with medical complexity, user preferences and needs, suggestions for improving current technologies, telehealth experiences during the COVID-19 pandemic, and design recommendations for new technologies. We did ask probes when necessary to capture more details in unbiased ways. The audio interview was over Zoom per institutional review board requirement, so we did not record the video or observe any nonverbal cues. Each interview lasted from 45 to 60 minutes, and the participants’ comfort level determined the duration in continuing to disclose their perceptions and share their experiences.

### Data Analysis

The interviews were recorded, transcribed, and analyzed using inductive thematic analysis. The raw data were initially labeled into themes to capture essential data. The themes emerged upon comparing experiences views, situations, and contexts from the same and different participants, and we gradually refined the coding schema. We emerged themes by comparing experiences, views, situations, and contexts from the same and different participants and gradually refined the coding schema. The first author (FE) coded the data and created the themes and a codebook, which the second researcher (OA) validated and refined. Both authors performed the coding using Excel (Microsoft Corp).

During the data analysis process, the authors used an inductive approach to identify themes and understand technological needs, preferences, available options, challenges, and suggestions for improvements. The inductive approach is characterized by a search for patterns [16]. We identified 4 primary themes with their codes. After coding the data, both authors collaborated to categorize the data within each theme into subthemes. Subthemes define common dimensions within the main theme [17]. The objective was to explore the data beyond identifying common or dominant themes to uncover unique insights [18].

### Ethical Considerations

This qualitative study used semi-structured interviews to collect data after obtaining ethics approval (ID 2023-006 (N)) from the Stevens Institute of Technology. The data was de-identified and does not include any identifier. Participants were paid 30 dollars compensation upon completion of the interviews.

## Results

### Overview

**Figure 1:**
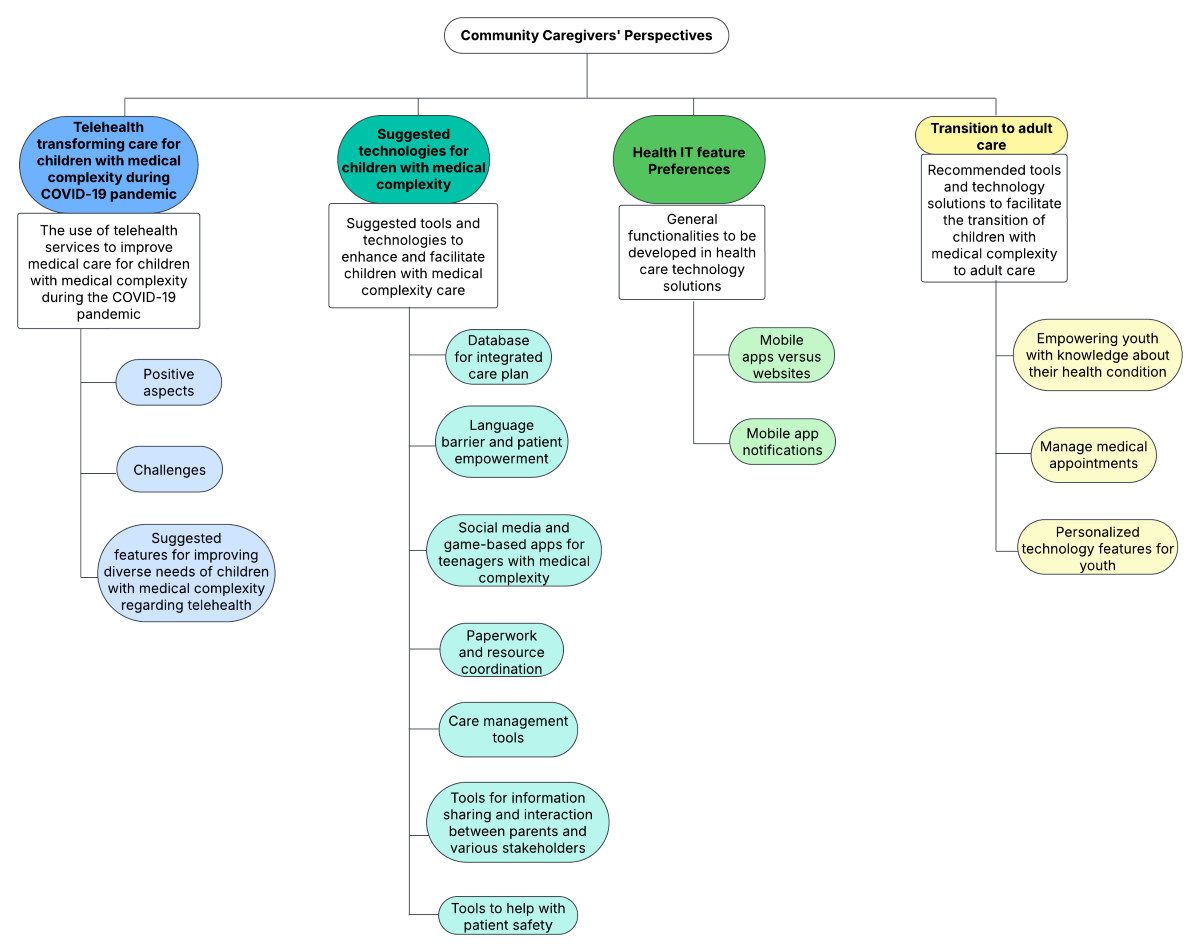
Identified major themes and subthemes.

The inductive analysis resulted in four major themes: (1) telehealth transforming care for children with medical complexity during the COVID-19 pandemic, (2) suggested tools and technologies for care for children with medical complexity, (3) HIT feature preferences, and (4) transition to adult care. Figure 1 shows the 4 major themes and associated subthemes.

### Theme 1: Telehealth Transforming Care for Children With Medical Complexity During the COVID-19 Pandemic

The first primary theme identified from the thematic analysis was telehealth use, which is defined as using telehealth services to improve medical care for children with medical complexity health needs during the COVID-19 pandemic. We also developed several subthemes for positive and negative perceptions regarding telehealth use in care for children with medical complexity and suggestions to improve telehealth technologies for care for children with medical complexity from caregivers’ perspectives.

The diverse needs of children with medical complexity result in varying health outcomes from telehealth services, depending on the child’s medical condition during the COVID-19 pandemic. For example, participant 9 reported improvements in the child’s health through telehealth visits, while participant 10 expressed dissatisfaction with the health outcomes achieved through telehealth therapy sessions:

They are all so different. I have one parent that I know when they went virtual, it worked beautifully with her daughter, who has limited speech. It helped so much because she was able to navigate everything. She had clear expectations. She is very visual. So it actually helped a lot.

Participant 9

Occupational therapy, or the physical therapy or speech therapy, even some of these therapy sessions cannot be offered for children who have very limited understanding or limited functional skills so having those virtual sessions were not beneficial for them, or meaningful for them. For example, if the child cannot follow a direction because of their cognitive ability, or because of that developmental status, then the virtual therapy is not meaningful for them. If the physical therapist is giving the child this direction but then the child cannot move, so the parent has to be right next to the child so that they can either assist the child or they can model that to the child. So then it is a barrier. If the parents are busy with their work, or if they have other commitments then the child is not getting the meaningful therapy sessions.

Participant 10

However, the participants who are the community caregivers shared common points regarding positive aspects, challenges they observed children with medical complexity parents experiencing, and ideas for improving telehealth services. Multimedia Appendix 1 presents subthemes of the positive aspects of telehealth as perceived by participants, alongside the challenges and limitations they experienced. It also includes suggestions for improving telehealth services to address the diverse needs of children with medical complexity. We reported at least 1 sample quote for each identified subtheme. Positive aspects include just-in-time access to care services (telehealth provides easier access to a variety of needed health care providers), facilitating physician-patient or parent interaction (telehealth facilitates interaction and communication between parents and providers during the COVID-19 pandemic), and overcoming geographical barriers (mitigating the barriers in health care services resulting from physical distance). However, participants also identified several challenges, including technical difficulties (the challenges and issues related to the technology used for telehealth appointments that impact the accessibility of services), a lack of communication while transitioning to telehealth during COVID-19 pandemic (the difficulties experienced due to unclear communication while shifting to telehealth services during the COVID-19 pandemic), and simplicity for non–tech-savvy parents (the need for technology that is easy to use and navigate, especially for parents who are not comfortable using various digital tools). In addition, participants suggested several features to improve telehealth for children with medical complexity, including visual graphics (using visual elements to enhance communication and engagement during the telehealth session), educational components (enhancing users’ understanding of telehealth), video and audio support (the critical need for high-quality video and audio technology in telehealth to facilitate effective and accurate communication between health care providers and patients), questionnaire addition (the integration of questionnaires into telehealth services), and providing variety of ways to communicate (offering diverse methods to facilitate communication for patients who may find it challenging to express themselves).

### Theme 2: Suggested Tools and Technologies for Care for Children With Medical Complexity

This theme covers suggestions for developing tools and technologies to enhance and facilitate care for children with medical complexity. Participants suggested developing various tools and technologies to support themselves or the parents of children with medical complexity, along with the features to be added to existing technology, as shown in Multimedia Appendix 2. One suggestion was the creation of a database for integrated care plans, a comprehensive system to help families manage and coordinate all aspects of their child’s health care. This would serve as a centralized database to provide a clear and organized record of their child’s information:

The parents need to keep records and, when anything changes, be ready and clear on what needs to happen. Even if a home nurse is coming to assist the parents, the parents are still, as I like to refer to them, the captain of the ship. They are the ones who need to know everything there is to know about their child’s health.

Participant 12

Participants also recommended tools to address language barriers and empower patients, aiming to overcome communication challenges between parents and health care providers arising from differences in languages or communication styles:

It becomes even more complex for my underserved families. For example, if the nurse needs to talk to the child’s doctors because the parent does not speak English, the parent should still be included in all of those conversations.

Participant 5

Some of the medical terminology is very difficult to translate into the parents’ native language. Although we have the technology in place, certain medical terms cannot be translated or interpreted in a way that parents can understand.

Participant 7

To enhance social development for teenagers with medical complexity, suggestions included social media and game-based apps designed to improve social skills and provide relevant health care information in engaging formats:

When things are developed in a very controlled setting, they work fabulously. However, as they trickle down into real life and society, and real people use them, often the system itself does not work as it should. For example, a system that allows communication without speech by exchanging pictures might help children, but instead of encouraging language development, it may lead to them becoming too comfortable using pictures. When applied in real time, this is very challenging for them, and they are not motivated to become verbal. I have heard many parents complain about this. These should be considered social skill lessons. If a game or solution could be developed and applied to real life, that would be great.

Participant 11

Paperwork and resource coordination tools were also highlighted to help families organize and track paperwork and resources associated with their child’s care:

Sometimes families are not always that organized. One of the things we always advise families is to write down information when they go to the doctor’s office, whether in person or virtually. We encourage them to learn how to communicate effectively with doctors and schools and to centralize their paperwork in one place. Managing all the documents, doctor’s notes, and medication records can be overwhelming, so it is important to teach families how to keep everything organized.

Participant 3

Regarding care management tools, participants suggested tools to assist parents with scheduling, tracking appointments, and managing medications:

Medications themselves can be particularly difficult to manage. Understanding and managing prescriptions, including knowing when to refill medications. There are complex instructions like “do not give this one with that one,” “take this one with food,” “this one needs to be refrigerated,” and “this one is an intramuscular injection.” With so many different medications and dosages, it can be overwhelming. For example, the same medication might require two pills in the morning and only one at night, which can change on the same day.

Participant 3

In addition, suggestions were made for tools to facilitate the exchange of information between parents, medical providers, and other care providers. Effective communication is essential for informed care coordination and comprehensive childcare:

Text-like feature that allows parents to choose who they want to include in a conversation. Parents do not necessarily want the school involved when it comes to medical matters. But it could have that option where the parent can choose to include people. It should have easy access to sharing and being able to pick and choose what you share and with whom. Making sure that the parent has control and is aware of who is getting what information.

Participant 9

Finally, the participants suggested tools to minimize risks and prevent errors in the care of children with medical complexity:

I think there is a need for tools and features that support children with medical complexity care to minimize risks. Unfortunately, families are sometimes the care managers. So, for example, if a child needs a diet for a gastrointestinal condition, yet also has kidney disease. They would need to consult with the medical team before they could give that diet and often that does not happen. The family has to say that this child has this medical condition and has to coordinate care between different teams, and it should not be that way, particularly with the availability of electronic health records. But unfortunately, they do not necessarily read the charts, or they are so siloed that they are only focused on their specific specialty.

Participant 5

### Theme 3: HIT Feature Preferences

The analysis showed another major theme around general recommendations preferred in HIT. This theme may assist system and software engineers in developing effective platforms that meet the needs of parents and supporters of children with medical complexity. Understanding these shared preferences is essential for designing technology that provides meaningful support and enhances engagement. The participants expressed their preferences for mobile apps and websites. They valued the convenience of mobile apps for quick access. In addition, mobile apps are beneficial for caregivers or patients with disabilities. However, they recognized that older adults caring for grandchildren with medical complexity prefer websites for their ability to display information on larger screens, such as laptops. The participants also emphasized the importance of mobile app notifications in coordinating care by providing timely reminders for caregivers. Table 1 presents the participants’ perspectives on mobile apps and websites.

**Table 1:** Perspectives on mobile apps and websites.

Subthemes and definitions	Quotes
Mobile apps versus websites (the comparison between mobile apps and websites in terms of preferences)	“Mobile access provides much more freedom. You do not need to have your laptop with you; if you are outside, you can still access data through your mobile device. However, many grandparents who take care of their grandchildren might not be able to have or use mobile apps.” [Participant 10] “I think it is important to have something that is not so cumbersome, something they can pull out and use, which is a phone. The invention of the smartphone might have so many capabilities for individuals with disabilities.” [Participant 12]
Mobile app notifications (the importance of mobile app notification feature for caregivers)	“I would like an app to coordinate everything and also send reminders or share reminders of what is coming up next. For instance, in terms of care coordination, I am adding information to the app, perhaps that my child visited a specialist on this date, then maybe the app can add reminders in additional ways about an upcoming appointment. Or maybe, if I say in my notes that I met with this specialist and need to schedule a follow-up appointment with so and so. Maybe the app can understand the action items from those notes and say you mentioned this, and you need to schedule a follow-up appointment with this specialist.” [Participant 1]

### Theme 4: Transition to Adult Care

The last primary theme focuses on a sensitive period in the continuum of care of children with medical complexity. At the age of 21 years, children with medical complexity experience the transition or transfer of care from pediatric settings to an adult setting, findings showed suggestions for technologies specifically for this transition period. The community caregivers highlighted several features of these technologies, especially those related to enabling self-care and management for the patients. The theme emphasized the necessity of designing technology to empower the youth with the necessary knowledge during this transition to adulthood. In addition, given the shortage of resources and skillsets in adult settings compared to pediatric settings for the children with medical complexity population, they highlighted the various needs children with medical complexity teenagers or young adults with medical complexity might have during and after the transition; therefore, a personalized technology, specifically addressing their needs, would be beneficial. Table 2 presents the subthemes related to various suggestions for using technology to facilitate the transition to adult care.

**Table 2:** Suggested technology to support the transition to adult care.

Subthemes and definitions	Quotes
Empowering youth with knowledge about their health condition (providing youth with the necessary knowledge to understand and manage their own health issues)	“A youth app would be so important to support health care transition. It is supposed to start at 12 because our kids are going to grow up. Youth are going to go to college, and they are going to have a new provider who is a specialist. Mom is not going to be there with them. So, I believe it is important to provide them with resources about their disability, what it is, and their medications.” [Participant 5]
Manage medical appointments (teaching youth with medical complexities how to schedule and organize their own health care visits)	“I think of an app that would help the child to make appointments such as a scheduler. As a mother of a child with medical complexity, we do everything. The truth is that they are going to grow up, and the first steps might even be just having your child sign their name when they come into a doctor’s office or calling to make an appointment. The app should be integrated with text to talk for youth with medical complexity.” [Participant 5]
Personalized technology features for youth (technology solutions that meet the unique needs and abilities of children with medical complexity)	“An app should be able to assess whether users can read well; based on this assessment, we would use either tenth or twelfth-grade English. If users have receptive issues or dyslexia, we will avoid using this font and instead use one without unusual markings. For example, consider a twelfth-grader who reads at a third-grade level but is transitioning to adulthood. Currently, there are no resources for this, so the child will often rely on information from his parents. This can be a real struggle because, although the content is intended for him, he does not have access to it.” [Participant 10]

## Discussion

### Principal Findings

This qualitative study explores formal community caregivers’ perspectives on integrating HIT in the care of children with medical complexity. Community caregivers are an essential part of the care model for children with medical complexity from underserved populations [13,14]. Our findings showed the critical benefits of telehealth for this population, especially during the COVID-19 pandemic, though some negative experiences and improvement suggestions are also noted. We also identified various specific needs that can promote future technology design to address the specific needs of children with medical complexity in care management as well as the interaction between the parents of children with medical complexity and care providers. Finally, our findings address the need for technologies that support the transition from pediatric to adult care for the children with medical complexity population. To our knowledge, this is the first study capturing children with medical complexity community caregivers’ perception of the role and needs of HIT in an effective care for children with medical complexity model.

### Shifting From In-Person Visits to Telehealth

The COVID-19 pandemic has accelerated the adoption of telehealth into the American health care systems, providing safe access to medical care [19,20]. The data reveal that telehealth provided alternatives and safer options to the children with medical complexity population during the peak time of the COVID-19 pandemic as a cost-effective solution that can potentially improve health outcomes for children with medical complexity, facilitate physician-caregiver interaction, and increase caregiver satisfaction, as reported in other studies [21-23]. Community caregivers emphasize that telehealth eases the burden on parents, especially those from underserved populations, by reducing the time they need to take off work and travel long distances, as discussed by other studies [24,25]. During the COVID-19 pandemic, telehealth was the safest method to maintain physical distance while ensuring timely and affordable care [26]. Telehealth facilitated follow-up care, reducing the burden on care teams and decreasing exposure risks for both patients and health care providers [27]. However, the findings also showed that there are certain tasks, especially in inpatient settings, which cannot be done using telehealth. Clear communication between parents and health care providers is needed to ensure timely and reliable care [28]. Our study also revealed that community caregivers advocate for the continuity of telehealth services after the COVID-19 pandemic, driven by the factors mentioned earlier. Despite its advantages, our analysis also showed several challenges related to telehealth use in the care of children with medical complexity. These challenges include technical difficulties, connection issues, a user-unfriendly interface, and a lack of clear instructions for parents to connect to the system [29-31]. These challenges should be addressed and enhanced for better use of telehealth in care for children with medical complexity.

A previous study focused on providing recommendations to enhance telehealth functionalities and support diverse communication needs from the perspective of children, which aligns with our findings [32]. Our participants reported that children with medical complexity often struggle to sit still, listen, and engage throughout a long telehealth session. They suggested that for children with medical complexity, particularly those with developmental disabilities, integrating fun graphics with timers into telehealth can transform their experience. This approach allows families to create a structured environment that actively engages children and leads to more productive telehealth sessions. Moreover, our participants suggest accommodating diverse communication preferences during telehealth sessions, as some children might feel more comfortable expressing themselves through text. Integrating features such as fill-in-the-blank sentences, text-to-voice options, and voice-to-text options can significantly enhance accessibility. In addition, allowing children to choose their voice gender and use emojis to convey their pain levels or express agreement and disagreement can facilitate more effective communication. These adaptations can help create a more inclusive and supportive telehealth experience.

In addition, health care providers must listen to parents’ concerns and answer their questions, as it will assist them in coping with difficult situations [33]. Our participants recommend that preappointment questionnaires may enhance communication between parents and health care providers. This approach will help parents in addressing their concerns in advance, which will be discussed during the session. In addition, integrating an educational component into telehealth will enhance parents’ understanding of the medical information presented during the session.

### Technologies for Improving Outcomes of Care for Children With Medical Complexity

Community caregivers suggested tools to address various needs of this population, including parent or patient empowerment, information management, care coordination, communication, and patient safety. Visiting the physician’s office can be stressful for this vulnerable group due to the unfamiliar environment [34,35]. Our study revealed that games and social media platforms could improve the social skills of children with medical complexity, such as interacting with the health care staff. Participants suggested gamified learning apps that focus on teaching what is socially appropriate and inappropriate. In addition, social media platforms, such as TikTok, Snapchat, and Instagram, provide short videos that capture children’s attention. This feature can help children with medical complexities learn how to interact in a physician’s office and encourage them to participate actively in their health management.

Several subthemes emerged from the analysis, showing various management tools to help the parents of children with medical complexity at different levels of care for children with medical complexity, not only in hospitals but mostly in home settings. The data clearly show the increasing interest among caregivers in integrating various HIT tools into the care for children with medical complexity journey to improve the quality of care. As this interest expands, concerns arise regarding validating these technologies, as many lack regulatory approval. In addition, improving patient safety in complex pediatric health care requires providing families with tools that help them with decision-making and communication. Successful communication requires timely information exchange between primary care, specialty care, and families to enhance medical outcomes for children with medical complexity [36]. Our participants indicated that parents often felt responsible for sharing information, as they are the primary drivers of their children’s care. Miscommunication and the use of multiple medications in complex medical conditions increase opportunities for error, particularly as children transition between health care settings and practitioners [37]. Communication barriers often prevent parents from properly filling prescriptions or adjusting dosages, resulting in frequent errors and increased risks to patient safety [38]. In addition, parents need to find the right channels and have a road map to assist in understanding and managing the complicated situations or challenges they will encounter in the future [39]. Therefore, community caregivers ask for effective technologies and tools to be developed to address these critical needs of the parents of children with medical complexity.

Another subtheme highlighted the need to address language barriers and empower parents and patients, especially those from underserved populations with low education levels. Parents with limited English proficiency encounter communication challenges that negatively affect access to and quality of health services for children with medical complexity [40]. Language barriers and health literacy limitations could be associated with parents’ less understanding of diagnoses, treatment options, care plans, and follow-up recommendations [41]. In addition, language barriers include a limited understanding of medical terminology while explaining the child’s diagnosis and treatment plan [42]. The parents want clear instructions, simplified medical terms, and concrete medication directions [43]. A well-designed tool can help these parents mitigate these barriers and become empowered in the care of their own children with medical complexity.

### Preferences and Specifications for User-Centric Design

The data showed some discussions on preferences on the type of technology platforms discussed by community caregivers. The participants discussed their preferences for mobile apps and websites, focusing on how these platforms can meet diverse needs. Smartphones allow users to access information and services anytime and anywhere [44]. However, older adults caring for children or grandchildren with medical complexity might prefer using desktops and laptops, as larger screens provide a more accessible information display.

The participants also emphasized the importance of reminders and notifications in managing their caregiving responsibilities effectively. Reminders are an important feature in mobile apps, helping users manage medication, appointments, and adherence to medical conditions [45]. For instance, the participants in our study mentioned that writing notes is a common practice to keep track of important tasks and appointments; however, this can often lead to information overload and disorganization. It was suggested that an app could analyze these notes and identify action items. Then, the mobile app could send reminders on upcoming appointments and other important tasks. This could be achieved using an artificial intelligence (AI)–powered mobile app that uses natural language processing to analyze the text notes. AI-assisted mobile apps could be designed to provide personalized experiences for parents and offer insights that drive better decision-making [46]. In addition, AI-assisted apps could be extended to assist parents in enhancing medication adherence by recognizing medications and verifying their ingestion [47].

### The Role of Technology in Supporting Transitions to Adult Health Care

Our data revealed an interesting theme focusing on the care transition or transfer of this vulnerable population from pediatrics to adult care and how to make this transition better by using various technologies and tools. These tools should educate youth about their complex medical conditions, medications, and overall health management [48]. Children with medical complexity will inevitably grow up, and they will need to navigate independent adult life and responsibilities. Managing multiple medical appointments and follow-ups becomes a significant skill while transitioning to adult care. Our participants suggested an app that includes features such as appointment scheduling and text-to-speech integration, which could help youth with medical complexity learn to navigate this aspect of their care independently. Furthermore, personalized technology solutions that adapt to individual reading levels and cognitive abilities are essential [49]. As suggested by Statewide Parent Advocacy Network participants, an app that provides content accommodating varying reading skills can ensure that children with medical complexity with different learning needs can equally engage with and access adult transition resources.

As shown in the literature, this transition period is risky, and adult settings are significantly less prepared compared to pediatric settings to care for this vulnerable population [50]. Transitioning from pediatric to adult health care is unique to each child and ideally occurs between the ages of 18 and 21 [51]. The negative impact of poorly managed care transition contributes to family burden and distress [52,53]. Families face significant challenges in motivating their children and often lack clarity about the steps needed for the adult care transition phase [53]. There is growing interest in leveraging HIT to facilitate the transition from pediatric to adult care [54,55]. Health care providers and parents support youth in developing autonomy, decision-making skills, and self-management by creating tailored plans that address their abilities and complexities [49]. This process can be further empowered by leveraging HIT, which can provide tools and resources to enhance personalized care and support this transitioning phase, as highlighted by the participants in our study.

### Limitations

A limitation of this study is that the interviews were conducted with members of only one organization. In addition, we only collected gender as demographic information about participants. Our participants were predominantly female, so future studies could balance the insights by adding more male community caregivers.

### Conclusions

This study explored and analyzed the needs of children with medical complexities and their caregivers throughout their care journey from community caregivers’ perspectives. We identified several areas where HIT could enhance care for children with medical complexity conditions. There is a need for improvement in telehealth and the development of mobile health apps across various areas of care for children with medical complexity, such as data management, educational resources, care coordination, and transition to adult care. By addressing these areas, technology designers can contribute to more effective, coordinated, and personalized care for children with medical complexity. This improvement will potentially lead to better health outcomes and a higher quality of life for children with medical complexity.

## Appendix

Multimedia Appendix 1 Telehealth subthemes, definitions, and sample quotes.

Multimedia Appendix 2 Suggested tools and technologies to be developed for children with medical complexity care.

## Abbreviations

AI: artificial intelligence
HIT: health IT
