# Supplementary material for: Community Caregivers’ Perspectives on Health IT Use for Children With Medical Complexity: Qualitative Interview Study
Source: JMIR Pediatr Parent. 2025 Feb 10;8:e67289. doi: 10.2196/67289 (PMC11851040; doi:10.2196/67289)
Supplement: Multimedia Appendix 1 [file pediatrics_v8i1e67289_app1.docx]

**Multimedia Appendix 1**. Telehealth subthemes, definitions, and sample quotes.

| Subtheme categories and subthemes *(definitions)* | | Quotes |
| --- | --- | --- |
| **Positive aspects** | | |
|  | Just-in-time access to care services *(telehealth provides easier access to a variety of needed providers)* | - “Telehealth gives increased access because there is a shortage of pediatric specialists.” [Participant # 4] |
|  | Facilitating physician-patient/parent interaction *(telehealth facilitates interaction and communication between parents and providers during the COVID-19 pandemic)* | - “Providers shared that telehealth was a boom to them because they could continue to support families during the Covid period. Even prior to Covid, I think there was an urgent need for telehealth. Professionals and even a lot of families are thankful that telehealth opportunities were made and both of them felt that telehealth was helpful.” [Participant # 1] - I think it made things a lot easier unless the child needs to be physically examined or requires lab tests. It is better if parents can use telehealth, as this avoids putting the child with medical complexities at risk of COVID-19, while still allowing them to receive the necessary care from all the specialists.” [Participant # 4] |
|  | Overcoming geographical barriers *(mitigating the barriers in health care services resulting from physical distance)* | - “I think there are times, especially when families have multiple children, it is hard for them to get to that doctor’s appointment, and they may run into transportation issues. Sometimes the disability transportation is not in that area, or the type of family care Medicaid HMO does not cover the transportation cost because you can get transportation, but it may not be within their plan.” [Participant # 3] - “There is a family that goes one or two days overnight in Philadelphia every month to do their doctor’s visits and if that family could just do half of those visits through telehealth, it would be so much easier. But they rescinded the telehealth support, and it is really hard to cross state lines now. It is very difficult if your child’s immune compromised and has such complex needs. It makes more sense to do some of them virtually than in person.” [Participant # 8] |
| **Challenges** | | |
|  | Technical difficulties *(the challenges and issues related to the technology used for telehealth appointments that impact the accessibility of services)* | - “When the time of the appointment came, and I clicked on the link, it did not work, and I did not have a number to call, or even when I called it, I was put on hold, or I was asked to leave a message, and they would call me back. But then all that troubleshooting that needed to happen, did not happen, and it looked as if I missed the appointment because the provider was on, and mine was not getting connected. So those types of things should be considered, the troubleshooting options. So, families are not frustrated waiting for appointments or getting missing appointments. With CMC, those appointments are very critical, and it is important to make sure that they are informed and provided the support they need.” [Participant # 1] - “Families may have a list of questions that they need to ask if the person setting up the telehealth does not really give them the instructions. Because some places want you to use a tablet or a laptop versus a phone. Families might ask if we have just a phone, is that okay? and then they might ask certain questions about their bandwidth and all those pieces. So, giving them resources to make sure that the patient is connected to the telehealth correctly before the meeting with the doctor, and to test telehealth before the appointment.” [Participant # 3] |
|  | Lack of communication while transitioning to telehealth during the COVID-19 pandemic *(the difficulties experienced due to unclear communication while shifting to telehealth services during the COVID-19 pandemic)* | - “I was arguing a bill for a family switching to telehealth. It was not as easy as people would think. I had to fight for a couple of years with a bill because they kept billing it wrong. I mean, there is a specific rate for telehealth and they were billing the full visit. As a parent when you are struggling with many other things, you are taking my time. I am spending my time that I need to help my children and do my job so arguing over bills every month becomes exhausting, and it is like another thing added to everything we already do.” [Participant # 9] - “They were trying to force telehealth but I need someone to draw blood. So, the miscommunications in that way of what gets funneled into telehealth versus what gets funneled into an in-person visit caused delays and confusion, and then, of course, just frustration over the whole matter.” [Participant # 11] |
|  | Simplicity for non–tech-savvy parents *(the need for technology that is easy to use and navigate, especially for parents who are not comfortable using various digital tools)* | - “The challenge was that not every office uses the same system. Some of them are more user-friendly. For example, anything on Zoom is like a piece of cake for me. However, when I have to use Google Meet, I often find myself wondering, where is this feature?” [Participant # 9] |
| **Suggested features for improving the diverse needs of children with medical complexity regarding telehealth** | | |
|  | Visual graphics *(using visual elements to enhance communication and engagement during the telehealth session)* | - “Sometimes you are sitting there waiting for the doctor to show up on telehealth, and the longer you wait, the more challenging it becomes for children. If you can have the child engage in another activity, as long as they are able to pause it when the doctor needs to talk to them. Many children on the spectrum or with intellectual or developmental disabilities benefit from visual cues. Incorporating visuals, like messages and engaging graphics, into the telehealth app could be helpful. For example, the app could display a message saying, ‘You only need to wait here for 5 minutes and work with me on your goals.’ and once the 5 minutes are up, the screen could show some fun graphics.” [Participant # 4] |
|  | Educational components *(enhancing users’ understanding of telehealth)* | - “I would emphasize adding the education component to telehealth. This would help families understand what to expect in the telehealth appointment, how they can get prepared for it, and how they can follow up if they have questions. Those types of add-ons would be great.” [Participant # 1] |
|  | Video and audio support *(the critical need for high-quality video and audio technology in telehealth to facilitate effective and accurate communication between health care providers and patients)* | - “High quality, video and audio, and clear communication because for accurate diagnosis and treatment both the parties need to have high-quality video and audio support.” [Participant # 10] |
|  | Questionnaire addition *(the integration of questionnaires into telehealth services)* | - “There should be a set of questions sent to parents to complete, addressing their concerns, what they want to achieve, and what brings them to the telehealth appointment. So there has to be some preparation before. And there has to be some follow-up after, not necessarily relying on that appointment to get all the information.” [Participant # 6] |
|  | Variety of ways to communicate *(offering diverse methods to facilitate communication for patients who may find it challenging to express themselves)* | - “I am thinking about my daughter, she has a medical complexity. She is a really good reader, but she sometimes struggles to find the right words to articulate her thoughts. For example, using fill-in-the-blank activities could help. One option might be something like, ”Today I feel ___,” with choices such as “happy,” “sad,” “mad,” and a space for her to type another word if needed. This approach allows her to communicate more easily if she has difficulty finding very specific words. Another option could be allowing her to communicate by typing freely in a text box, providing a variety of ways to express herself in a telehealth appointment.” [Participant # 11] |
