# Supplementary material for: Community Caregivers’ Perspectives on Health IT Use for Children With Medical Complexity: Qualitative Interview Study
Source: JMIR Pediatr Parent. 2025 Feb 10;8:e67289. doi: 10.2196/67289 (PMC11851040; doi:10.2196/67289)
Supplement: Multimedia Appendix 2 [file pediatrics_v8i1e67289_app2.docx]

**Multimedia Appendix 2**. Suggested tools and technologies to be developed for children with medical complexity care.

| Subthemes | Quotes |
| --- | --- |
| Database for integrated care plan *(suggestions for a comprehensive system that enables families to manage and coordinate all aspects of their child’s health care)* | - “There is a need for technology that can help families manage all the health records of their children and coordinate their care. It would be beneficial to have a system that maintains all the information in one place.” [Participant # 1] |
| Language barriers and patient empowerment *(suggestions to overcome language barriers that occur when patients or parents and health care providers have difficulty understanding each other due to differences in language or communication styles)* | - “We do not necessarily know all medical terminology. If there were a resource to help parents understand the child’s diagnosis, the terms being used, and the medications prescribed. It would serve as a resource library for parents to get educated, even though the internet is so wide, not all resources are trusted.” [Participant # 6] |
| Social media and game-based apps for teenagers with medical complexity *(suggestions for digital platforms designed to enhance social skills and provide valuable information through games and videos)* | - “Games and social media could be valuable areas for developing an app focused on social skills. Such an app should include lessons on what is socially appropriate and inappropriate. Also, I have noticed on TikTok and Instagram that the reel feature, short videos, effectively delivers information. What attracts kids is that these videos, ranging from one to three minutes, can be catchy and go viral. It would be beneficial to create short, helpful videos on how to interact in a doctor’s office. Additionally, on Instagram and Snapchat, the ability to send voice memos is very important for teenagers with disabilities. For example, my niece finds it easier to send voice memos rather than type out messages due to her medical complexity, the spinal cord injury.” [Participant # 9] |
| Paperwork and resource coordination *(suggestions for tools to help in organizing and tracking paperwork and resources)* | - “A user-friendly app that would help compile and organize paperwork, and by extension, SPAN can use something like that to compile resources available to parents in a categorized way. Because we offer training to parents on how to organize all of the paperwork, and all of the things they get from the school district, and the doctors.” [Participant # 11] - “It would be helpful to have an app as a guide for the parents. What are the best practices and where to gather information for their children? An app that parents can navigate, and it could give them guidance on what to track and what to keep so depending on the child’s needs.” [Participant # 9] |
| Care management tools *(suggestions for tools to help parents in scheduling and tracking appointments as well as managing medications)* | - “An app to manage scheduling whether it is special needs transportation, doctors, appointments, and just remembering when to refill prescriptions.” [Participant # 4] - “Having a history feature in an app would be great if it allowed parents to quickly access a list of all dates and view the schedule of appointments in one glance, or for a specific date. Additionally, a brief description could indicate the reason for each visit. Many parents manage care for multiple providers, making it challenging to track calendars, medications, and vaccines.” [Participant # 5] - “Parents are struggling in managing appointments. So, I would suggest an app being able to track multiple calendars in one setting, sending reminders, adding notes from previous appointments so they can be ready for the next visits and then maybe adding the vitals like this was the blood pressure or weight management or whatever data as it is individualized.” [Participant # 9] |
| Tools for information sharing and interaction between parents and various stakeholders *(suggestion for tools to facilitate the exchange of information between parents, medical providers, and other care providers to ensure coordinated and informed care for the child)* | - “I think if the family can document what their child’s strengths and needs are, what their child is allergic to; what their child has the capacity to do, etc. And reviews that with medical providers and other care providers for their child. I think the ability to share that information from an app with these providers and making sure that they have seen it. And somewhat acknowledging, and they send back an acknowledgment, stating that they have reviewed everything.” [Participant # 1] |
| Tools to help with patient safety *(suggestions for tools designed to minimize risks and prevent mistakes in childcare)* | - “There is a ton of stuff out there. There are things like fall alerts. There are Apple watches, heart rate detection, and those kinds of things. There are seizure detection devices. But none of them have been FDA approved so it is not reliable.” [Participant # 4] - “A tool for medication interaction so if there is a concern, we have to switch the medication. Especially in the specialists’ appointments. You do not want to miss that appointment for these follow-up questions for later which could be a concern so that tool would be helpful.” [Participant # 6] - “I know that right now we are using Alexa to send the reminder that it is time to take medicine, but the device giving the reminder needs to be improved. Things need to be like Alexa said that one time. But there is nobody to check if the child took the medicine or not, or how the child is administering the medicine.” [Participant # 10] |
